# Urea is a drop-in nitrogen source alternative to ammonium sulphate in *Yarrowia lipolytica*

**DOI:** 10.1016/j.isci.2022.105703

**Published:** 2022-12-01

**Authors:** Oliver Konzock, Simone Zaghen, Jing Fu, Eduard J. Kerkhoven

**Affiliations:** 1 Division of Systems and Synthetic Biology, Department of Biology and Biological Engineering, Chalmers University of Technology, Göteborg, Sweden

**Keywords:** Biological sciences, Microbiology, Applied microbiology

## Abstract

Media components, including the nitrogen source, are significant cost factors in cultivation processes. The nitrogen source also influences cell behavior and production performance. Ammonium sulfate is a widely used nitrogen source for microorganisms’ cultivation. Urea is a sustainable and cheap alternative nitrogen source. We investigated the influence of urea as a nitrogen source compared to ammonium sulfate by cultivating phenotypically different *Yarrowia lipolytica* strains in chemostats under carbon or nitrogen limitation. We found no significant coherent changes in growth and lipid production. RNA sequencing revealed no significant concerted changes in the transcriptome. The genes involved in urea uptake and degradation are not upregulated on a transcriptional level. Our findings support urea usage, indicating that previous metabolic engineering efforts where ammonium sulfate was used are likely translatable to the usage of urea and can ease the way for urea as a cheap and sustainable nitrogen source in more applications.

## Highlights

• previous metabolic engineering efforts are likely translatable to urea
• cell physiology unaltered by urea versus ammonium sulfate
• transcriptome shows no significant concerted changes due to different nitrogen sources
• genes involved in urea uptake and degradation are not transcriptionally regulated

## Graphical abstract

**Figure:**
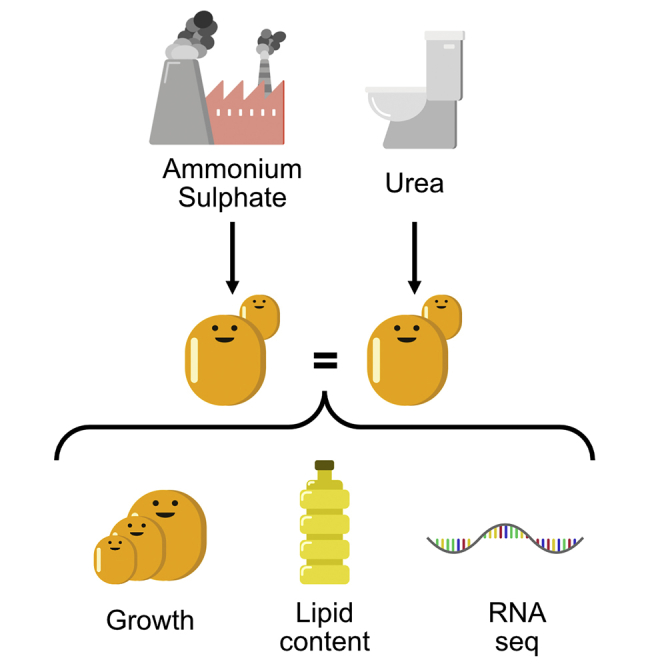

## Introduction

Over the past decade, nonconventional yeasts as host organisms for biotechnology applications have been on the rise. Advanced genetic engineering tools and decreasing prices for omics analysis allow more studies on non-model organisms. The oleaginous yeast *Yarrowia lipolytica* is one example of such an organism. The initial interest was focused on the outstanding lipid production capacity of *Y. lipolytica*,^1^^,^^2^ which can be increased to up to 90% of its dry weight.^3^ However, this host has also been shown to be suitable for non-lipid products, e.g. terpenoids such as carotenoids and polyketides like flavonoids.^4^ For the production of these molecules, fat-free strains have been engineered that lack the essential genes for storage lipid production.^5^^,^^6^^,^^7^

Oleaginous yeasts, such as *Y. lipolytica*, can accumulate at least 20% of their dry weight as lipids in wild-type strains. One of the primary triggers for lipid production is nitrogen starvation. Nitrogen limitation (high C/N ratio) induces adenosine monophosphate (AMP) depletion by AMP deaminase, which inhibits the isocitrate dehydrogenase of the tricarboxylic acid (TCA) cycle. This inhibition leads to an excess of citrate in the mitochondria, which is transported into the cytosol by malate/citrate transferase. In the cytosol, citrate is cleaved to form acetyl-coenzyme A (CoA) by ATP-citrate lyase and is further converted to fatty acids.^8^

Together with the carbon source, the nitrogen source is one of the main cost factors in the media of large-scale cultivation.^9^ The most widely used nitrogen source in microbial cultivation is ammonium sulfate, produced by sulfuric acid treatment of ammonia. Industrial ammonia production is mostly via the energy- and carbon-intense Haber-Bosch process that fixes nitrogen with hydrogen at high temperature and pressure.^10^

Urea is an interesting alternative that is more sustainable since it can be extracted from urine, and cost calculations showed a lower cost per mol nitrogen for urea than that for ammonium sulfate.^11^ In addition, urea can be produced from municipal solid waste in an economical and environmental way.^12^ In contrast to ammonium sulfate, urea utilization does not acidify the media, which requires less base addition during pH-controlled large-scale cultivation. However, changing the media composition can affect cell behavior and the production performance of microorganisms.^13^^,^^14^^,^^15^^,^^16^ In *Y. lipolytica*, the nitrogen source was also linked to dimorphic growth.^17^

To further understand the cells' reaction to different nitrogen sources, it is not enough to limit observation to single parameters (e.g., lipid content, hyphenation, and growth rate); we need to monitor the cell as a whole. One approach for studying cell behavior on a genome-wide level is transcriptomic analysis. Transcriptomics maps the abundance of all mRNA molecules of a cell (i.e. the transcriptome), thereby determining the total gene expression. Comparison between different conditions can then reveal changes in cell behavior.

In this study, we performed transcriptomic analysis of *Y. lipolytica* cultivations where we varied three variables: (a) we used three strains differing in their lipid accumulation ability; (b) cultivated in either carbon- or nitrogen-limiting conditions; (c) with urea or ammonium sulfate as nitrogen source. With this experimental setup, we aimed to investigate whether a response to the nitrogen source might differ depending on the amount of lipid accumulation.

## Results

**Figure 1:**
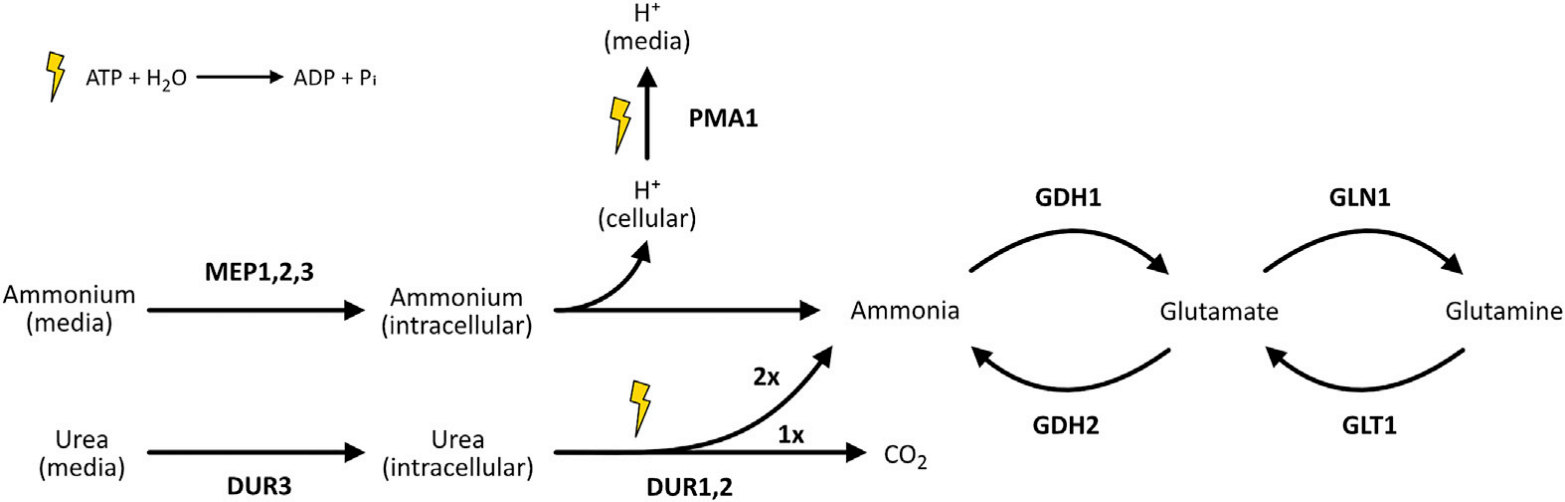
Comparison of ammonium and urea assimilation Ammonium is transported into the cell and deprotonates to ammonia which is further metabolized to glutamate and glutamine. The additional proton is transported out of the cell under the consumption of ATP. Urea is transported into the cell and converted to two ammonia and carbon dioxide, consuming one ATP.

From the *Y. lipolytica* genome, potential homologs could be identified to reconstruct the ammonium and urea assimilation pathways. The pathways are not dissimilar from the model yeast *Saccharomyces cerevisiae*, indicating a conserved mechanism, although the number of homologous genes occasionally differed (e.g. DUR3, Table S1). Briefly, ammonium and urea are transported into the cell by MEP1,2,3 and DUR3, respectively (Figure 1). Ammonium dissociates into ammonia, and the released proton is transported back into the medium by the plasma membrane H^+^-ATPase (PMA1) under the consumption of one ATP per proton.^18^ Urea is converted into two ammonia molecules by a urea amidolyase (Dur1_2). DUR1_2 is a multifunctional enzyme with urea carboxylase and allophanate hydrolase activity. The first activity is biotin-dependent and converts urea into allophanate under the consumption of one ATP and bicarbonate. The second activity consumes water and releases CO_2_ and two ammonia molecules, making this process more energy efficient than the usage of ammonium from the media.^19^ Ammonia is then incorporated into glutamate and glutamine by the NADP-dependent glutamate dehydrogenase (GDH1) and glutamine synthetase (GLN1), respectively. Glutamate and glutamine both serve as the starting points for amino acid production. In addition, glutamate can be converted back by the NAD-dependent glutamate dehydrogenase (GDH2) to ammonia and α-ketoglutarate, which links the nitrogen metabolism to the TCA cycle.^20^ The gene expression of both nitrogen pathways has been shown to be regulated by the available nitrogen source in *S. cerevisiae*.^21^^,^^22^

### Cell physiology does not change with urea compared to ammonium sulfate as a nitrogen source

We investigated the effect of urea versus ammonium sulfate as a nitrogen source in three different *Y. lipolytica* strains. The strain OKYL049 is a genetically engineered obese strain carrying DGA1 overexpression (YALI1_E38810g) and are1 deletion (YALI1_F09747g).^23^ The strain JFYL007,^24^ also referred to as Q4 strain, is incapable of synthesizing the storage lipid triacylglycerol or sterol esters due to the deletion of four genes, Δare1 (YALI1_F09747g), Δlro1 (YALI1_E20049g), Δdga1 (YALI1_E38810g), and Δdga2 (YALI1_D10264g).^5^ OKYL029^25^ has no modifications of the lipid production and displays a wild-type lipid phenotype. In all strains, hyphae formation was abolished by deleting MHY1 (YALI1_B28150g) to ease the bioreactor cultivations and ensure similar cell morphology despite different culturing conditions.^25^

We performed chemostat cultivations at a dilution rate of 0.1 in a minimal medium under carbon or nitrogen limitation (C/N ratio 3 or 116) with urea or ammonium sulfate as equimolar nitrogen sources. The pH was maintained at a value of 5 by the addition of potassium hydroxide. In contrast to ammonium sulfate, urea requires significantly less base addition during cultivation (Figure S1), which can further lower process costs.

The cell physiology was largely unaffected by the nitrogen source in our chemostat cultivations (Table 1). The nitrogen assimilation from ammonium sulfate has a higher cost (1 ATP per ammonia) compared to urea (½ ATP per ammonia). We reasoned that this might have an impact on the biomass, lipid content, their corresponding yields, or the specific uptake rate of glucose (r-Glucose). However, only the obese strain (OKYL049) showed significant changes in biomass in nitrogen limitation. The other strains showed no statistically significant changes (p value <0.01) in any of the measured parameters between ammonium sulfate and urea.

**Table 1:** Physiological changes of the strains in different C/N ratios and nitrogen sources

C/N ratio	N-source	OKYL029		OKYL049		JFYL007	
		3	116	3	116	3	116
Biomass (g/L)	AS	3.5 ± 0.2	2.0 ± 0.3	3.6 ± 0.1	2.2 ± 0.1^a^	3.5 ± 0.1	1.3 ± 0.1
	U	3.7 ± 0.3	1.7 ± 0.1	3.5 ± 0.1	1.8 ± 0.2^a^	3.8 ± 0.0	1.4 ± 0.4
Lipid content (%)	AS	2.7 ± 0.5	8.5 ± 0.6	2.5 ± 0.1	12.1 ± 1.6	2.4 ± 0.4	5.9 ± 0.9
	U	2.6 ± 0.3	9.8 ± 1.7	2.8 ± 1.0	10.2 ± 1.3	2.2 ± 0.1	7.8 ± 1.5
Biomass yield (gCDW/gGlucose)	AS	0.46 ± 0.03	0.48 ± 0.06	0.48 ± 0.01	0.36 ± 0.01	0.47 ± 0.02	0.44 ± 0.04
	U	0.50 ± 0.03	0.42 ± 0.02	0.47 ± 0.01	0.42 ± 0.04	0.51 ± 0.01	0.36 ± 0.10
Lipid yield (gLipid/gGlucose)	AS	1.2 ± 0.3	4.1 ± 0.5	1.2 ± 0.1	4.3 ± 0.6	1.1 ± 0.2	2.6 ± 0.7
	U	1.3 ± 0.2	4.2 ± 0.8	1.3 ± 0.5	4.3 ± 0.9	1.1 ± 0.0	2.9 ± 1.1
r-Glucose	AS	0.23 ± 0.01	0.22 ± 0.02	0.22 ± 0.01	0.29 ± 0.01	0.22 ± 0.01	0.24 ± 0.02
	U	0.21 ± 0.01	0.25 ± 0.02	0.23 ± 0.01	0.26 ± 0.02	0.20 ± 0.01	0.31 ± 0.10

Displayed is the mean ± SD of at least three replicates. Significance was calculated between the two nitrogen sources ammonium sulfate (AS) and urea (U), with a two-tailed homoscedastic t-test.

a Indicates a significant change (p value <0.01) between the ammonium sulfate and urea conditions.

To investigate whether the nitrogen source affects the fatty acid composition, we performed lipid extraction and converted the fatty acid chains of all lipids into fatty acid methyl esters (FAME). We then analyzed the distribution of the five most dominant fatty acids: palmitic acid (C16:0), palmitoleic acid (C16:1), stearic acid (C18:0), oleic acid (C18:1), and linoleic acid (C18:2) (Table 2).

**Table 2:** Changes in the strains' fatty acid composition (% of total fatty acid) in different C/N ratios and nitrogen sources

C/N ratio	N-source	OKYL029		OKYL049		JFYL007	
		3	116	3	116	3	116
C16:0 (%)	AS	9.5 ± 0.5	15.5 ± 1.5	9.0 ± 0.1	16.7 ± 0.2	8.4 ± 0.3	17.7 ± 1.1
	U	8.8 ± 0.2	14.3 ± 0.5	10.3 ± 2.7	13.6 ± 0.6^a^	8.3 ± 0.2	18.0 ± 0.4
C16:1 (%)	AS	7.8 ± 0.2^a^	7.2 ± 0.2	7.7 ± 0.7	5.8 ± 0.4	10.9 ± 0.5^a^	9.8 ± 0.8
	U	9.2 ± 0.2^a^	7.0 ± 0.6	8.6 ± 0.7	5.5 ± 0.2	12.4 ± 0.2^a^	10.5 ± 0.2
C18:0 (%)	AS	1.1 ± 0.2	6.0 ± 0.7	3.8 ± 0.1	10.4 ± 0.7	0.3 ± 0.1	1.7 ± 0.1^a^
	U	0.8 ± 0.1	4.7 ± 0.3	3.4 ± 0.4	9.6 ± 0.3	0.2 ± 0.0	2.2 ± 0.1^a^
C18:1 (%)	AS	49.9 ± 0.3^a^	47.9 ± 1.4	54.0 ± 1.9	54.1 ± 0.7	43.3 ± 1.0^a^	17.9 ± 0.5^a^
	U	47.7 ± 1.2^a^	49.6 ± 1.4	52.9 ± 2.3	54.4 ± 0.2	40.6 ± 0.3^a^	16.1 ± 0.3^a^
C18:2 (%)	AS	31.7 ± 0.7	23.4 ± 3.3	25.5 ± 1.4	13.1 ± 1.2^a^	37.1 ± 0.8	52.9 ± 0.1
	U	33.5 ± 1.2	24.5 ± 1.0	24.8 ± 3.8	16.9 ± 1.1^a^	38.5 ± 0.3	53.2 ± 0.6
Saturated/Unsaturated	AS	0.12 ± 0.01	0.27 ± 0.04	0.15 ± 0.00	0.37 ± 0.02^a^	0.10 ± 0.00	0.24 ± 0.02
	U	0.11 ± 0.00	0.23 ± 0.00	0.16 ± 0.03	0.30 ± 0.02^a^	0.09 ± 0.00	0.25 ± 0.01
C16/C18	AS	0.21 ± 0.01	0.29 ± 0.02	0.20 ± 0.01	0.29 ± 0.00^a^	0.24 ± 0.00^a^	0.38 ± 0.01
	U	0.22 ± 0.00	0.27 ± 0.02	0.23 ± 0.03	0.24 ± 0.01^a^	0.26 ± 0.00^a^	0.40 ± 0.01

Displayed is the mean ± SD of at least three replicates. Significance was calculated between the two nitrogen sources ammonium sulfate (AS) and urea (U), with a two-tailed homoscedastic t-test.

a Indicates a significant change (p value <0.01) between the ammonium sulfate and urea conditions.

The fatty acid composition is often of interest when *Y. lipolytica* is applied to produce lipid derivatives, e.g. food oils.^26^ In these cases, nitrogen starvation is used to trigger lipid production to achieve high lipid titers. Under nitrogen-limiting conditions, the obese strain (OKYL049) showed significant changes in C16:0 and C18:2 between the two nitrogen sources. In addition, we observed a significant change toward lower saturation and longer chain length (C16/C18) in urea compared to ammonium sulfate. The storage-lipid-free Q4 strain (JFYL007) showed significant changes in the C18:0 and C18:1, which did not result in an overall significant change in saturation or chain length of the fatty acids.

Under carbon-limiting conditions (C/N ratio 3), storage lipid production is not triggered and the three strains have a similar lipid content. Therefore, most of the extracted fatty acids are expected to originate from phospholipids (mostly lipid membranes) and free fatty acids. Under this condition, we observed a significant change between urea and ammonium sulfate in C16:1 and C18:1 for the strains OKYL029 and JFYL007. For the latter, we also found a significant change toward longer chain length in urea compared to ammonium sulfate. We did not observe any significant changes under carbon limitation in the obese strain (OKYL049) between the nitrogen sources. Overall, these results indicate that the fatty acid composition of the storage lipids (mostly triacylglycerol) can be slightly influenced by the nitrogen source, while the membrane and free fatty acid composition are not consistently affected throughout all three strains.

As a next step, we performed RNA-seq analysis to probe whether any transcriptional changes occurred that might influence the phenotype beyond our measured parameters.

### Genotype and C/N ratio dominate transcriptional changes

**Figure 2:**
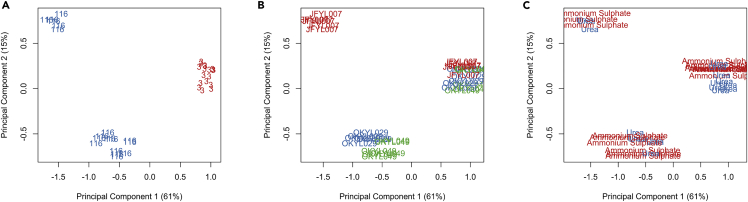
Principal component analysis (PCA) plot of RNA-seq samples The panels display the same PCA result, but samples are labeled differently based on either (A) nitrogen source, (B) C/N ratio, or (C) strain. PCA analysis was performed with log2 count per million, after removing low count genes and normalizing gene counts between samples with trimmed mean of M values (TMM) method.

A principal component analysis (PCA) was first performed to assess overall similarities and dissimilarities between the expression profiles of the samples (Figure 2).

In each of the conditions and strains, the replicates clustered well together, demonstrating the reproducibility of the experimental setup. We found that the nitrogen source (Figure 2A) only resulted in minor separation across the samples. Meanwhile, as the C/N ratio severely affected cell physiology (e.g. lipid content, Table 1), this also significantly separated the samples in the PCA, with PC1 explaining 59% of the variance. Cell physiology was further affected by the strain genotype, primarily in nitrogen limitation (C/N ratio 116), which was reflected in the triacylglycerol-free strain separating further away from the other two strains. In carbon limitation (C/N ratio 3), the strains showed only minor variance, indicating that the choice of nitrogen source has little effect on gene expression when available in copious amounts. Overall, these results showed that the C/N ratio resulted in the biggest differences between samples while the nitrogen source only attributed to minor variance.

### Gene ontology analysis revealed no coherent systemic response to the nitrogen source

To probe whether a systemic regulatory response could be observed, we performed gene ontology (GO) term analysis and compared the resulting GO terms in a heatmap to identify GO terms that are relevant in multiple strains and conditions (Figures S2–S4).

Similar to the results of differentially expressed genes, we only found a few GO terms that were identified in multiple strains and conditions. We did not find any GO term shared between all six comparisons (three strains and two C/N ratios). For carbon limitation, we identified three GO terms that were found in all three strains: protein-macromolecule adaptor activity, copper ion binding, and yeast-form cell wall. For nitrogen limitation, we identified two GO terms that were found in all strains: heme binding and aldehyde dehydrogenase (NAD^+^) activity. We could not directly link these GO terms to urea or ammonium sulfate. While various GO terms were seemingly somewhat enriched in some of the conditions, we could not deduce any coherent systemic response to the nitrogen source. Instead, we compared the differential expression of individual genes to catalog those that showed consistent regulation across strains and/or conditions.

### Urea is an alternative nitrogen source without a significant impact on the cell physiology or transcriptome

**Figure 3:**
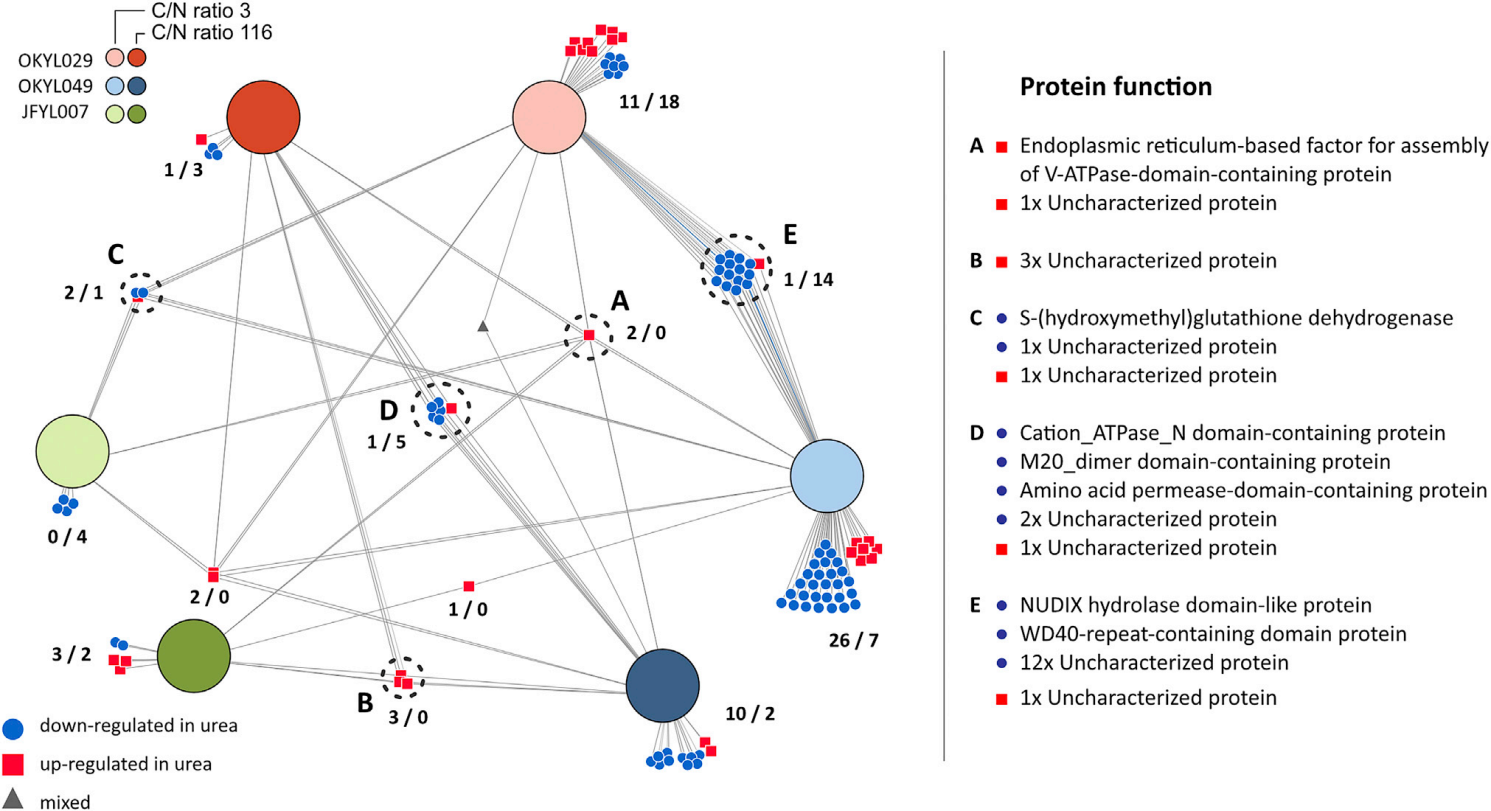
Visualization of overlap of differentially expressed genes in different conditions Displayed are the significant (adjusted p value <0.05) differentially (absolute fold change >1) expressed genes of each strain and C/N ratio in urea compared to ammonium sulfate. Numbers indicate the number of down-/up-regulated genes. Clusters A to E contain groups of genes of special interest as discussed in the results. The UniProt protein function of the corresponding genes is listed on the right-hand side. A full list including gene names can be found in Table S2. For visualization, the DiVenn web tool was used.^28^

We performed differential gene expression analysis to identify genes that are significantly (adjusted p value <0.05) differentially expressed (DE) (absolute fold change >1) as an effect of the different nitrogen sources and visualized them in a network plot (Figure 3). The most interesting clusters are circled, and their genes are listed in Table S2. A coherent response to nitrogen source irrespective of lipid accumulating capability would be represented by those genes that are DE in all strains and nutrient limitations (cluster A). Cluster A only contained two upregulated genes, of which YALI1_C01505g encodes a protein of unknown function, and YALI1_A01298g is an endoplasmic reticulum-based factor for assembling V-ATPase-domain-containing protein with similarity to VPH2 of *S. cerevisiae*, which is required for the assembly of the V-ATPase. This upregulation could reflect an increased need to acidify some cell organelles to maintain pH homeostasis,^27^ which could be linked to ammonium sulfate assimilation yielding one H^+^ per ammonia (Figure 1).

Since the C/N ratios had a significant impact on the overall gene expression (Figure 2B), we were also interested in the clusters of DE genes that shared a coherent response in all three strains under the same condition (carbon or nitrogen limitation) (cluster B and C). Cluster B only contained three uncharacterized proteins, and cluster C contained two uncharacterized proteins as well as an S-(hydroxymethyl)glutathione dehydrogenase (YALI1_F13170g).

As the Q4 strain (JFYL007) showed a very different behavior in contrast to the other two strains (Figure 2C and Table 1), we further inspected the clusters of genes that shared a coherent response across the more similar strains, OKYL029 and OKYL049, in the same conditions (cluster D and E). 16 of the 21 genes of clusters D and E were uncharacterized proteins. For the remaining proteins, we could not reconcile a coherent response to the change in nitrogen source.

In summary, the low variance identified in the PCA, the low number of DE genes, and the low overlap between them indicate that the nitrogen source (urea or ammonium) has minimal effect on the overall transcriptome.

### Genes associated with urea transport and degradation are not significantly induced by urea compared to ammonium sulfate

**Figure 4:**
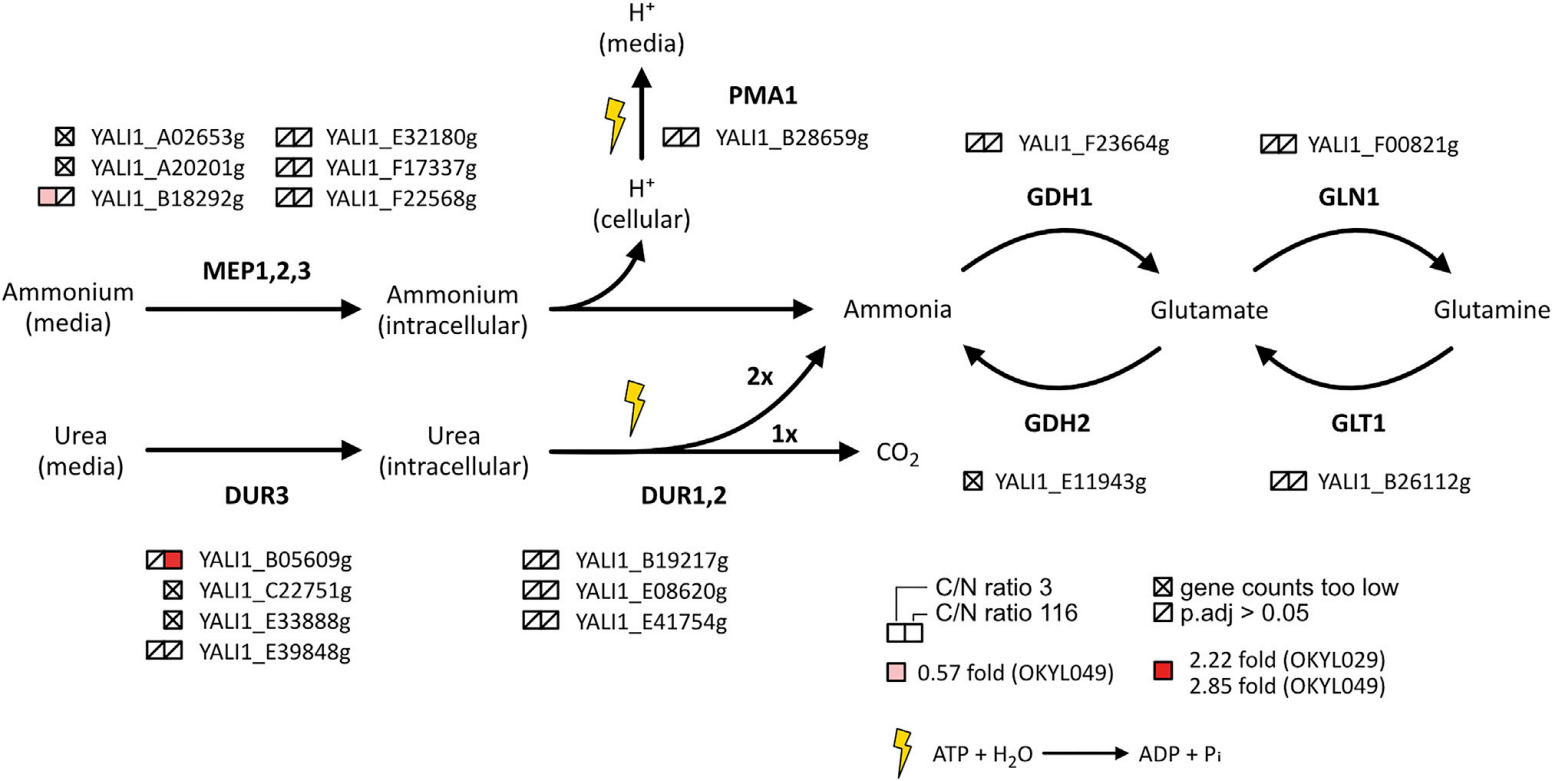
Influence of urea as nitrogen source compared to ammonium sulfate on the expression of the urea metabolic pathway genes Displayed are potential homologous *Y. lipolytica* genes and their expressional change when comparing the use of ammonium sulfate versus urea as a nitrogen source. The genes were identified from different sources, as listed in Table S1. Genes marked with x have been removed during the filtering of the gene counts; genes marked with/did not show any significant (adjusted p value <0.05) change between the nitrogen sources.

We further explicitly investigated if the expression of genes directly associated with urea metabolism are nitrogen source dependent (Figure 4). *Y. lipolytica* has three genes that are annotated as homologs of urea amidolyases (YALI1_B19217g, YALI1_E08620g, and YALI1_E41754g). However, none showed a significant (adjusted p value 0.05) different expression between the two nitrogen sources in either of the tested C/N ratios. There are four genes with homology to the *S. cerevisiae* urea transporter DUR3 (YALI1_B05609g, YALI1_C22751g, YALI1_E33888g, and YALI1_E39848g). Gene expression data from two of them (YALI1_C22751g and YALI1_E33888g) were removed during the filtering step because gene counts were too low (both have a lower protein sequence similarity to *Sc*DUR3 than the other homologs). Of the remaining two genes, we only observed a significantly different expression for YALI1_B05609g. Cultivation at C/N ratio 116 with urea instead of ammonium sulfate increased expression of this gene by 2.22- and 2.85-fold in the OKYL029 and OKYL049 strains, respectively. A similar trend was found for the ammonium transporter and ammonium permeases. Six genes have homology to *S. cerevisiae* MEP1,2,3. Also here, gene expression data from two of the genes were removed during filtering (YALI1_A02653g and YALI1_A20201g), while three other genes did not show any significant different expression (YALI1_E32180g, YALI1_F17337g, and YALI1_F22568g) in any strain or C/N ratio. Only YALI1_B18292g showed a modest 0.57-fold increase in strain OKYL049 in carbon limitation (C/N ratio 3). The V-ATPase PMA1 (YALI1_B28659g) that is mainly responsible for the regulation of pH homeostasis was not DE in any strain under any condition. None of the four genes involved in the glutamine pathway (YALI1_F00821g, YALI1_B26112g, YALI1_F23664g, and YALI1_E11943g) showed a significantly different expression.

These results indicate that none of the urea amidolyase, the urea or ammonium transporters, or genes of the glutamine pathway are induced by the presence of urea or ammonium on a transcriptome level in *Y. lipolytica*.

## Discussion

Most *Y. lipolytica* metabolic engineering studies have relied on the use of ammonium sulfate as a nitrogen source. While it can be argued that urea is an economically and environmentally attractive alternative nitrogen source,^12^ it would be detrimental if the cellular response to the use of urea would undermine the significant process that has been made to develop *Y. lipolytica* as a promising microbial cell factory. In that light, it has been reassuring to observe that a thorough analysis with diverse lipid phenotypes and cultivation conditions revealed no significant differences in the cell physiology or transcriptome upon the use of urea instead of ammonium sulfate. Previous studies have found significant differences in gene expression when using a simple nitrogen source (ammonium sulfate) and a complex nitrogen source (peptone plus yeast extract).^29^ We did not find major expression changes in our comparison of ammonium sulfate and urea, suggesting that the organic nitrogen source urea is very similar to the simple inorganic nitrogen source ammonium sulfate, and the difference in assimilation energetics does not majorly impact cell growth. Other studies that used urea as a nitrogen source in *Y. lipolytica* before did not report any growth differences^25^ or an improvement of growth in urea compared to other nitrogen sources.^11^ In addition, first experiments using synthetic and real human urine as nitrogen source for the cultivation of *Y. lipolytica* showed promising results with similar growth and biomass formations compared to ammonium sulfate, highlighting the future potential of urea as a nitrogen source.^11^

The minor changes observed in the fatty acid profile under carbon limitation (C/N ratio 3) most likely derive from changes in membrane fatty acids composition, changes that become visible when the contribution of the storage lipids (triacylglycerols or sterol esters) to the lipid content is very low. These changes could be a stress response aimed at adjusting membrane fluidity^30^ and connected to the acidification process of ammonium assimilation. However, we did not observe this change in the obese strain OKYL049. In addition, we did not find overall changes in the saturation or chain length of the fatty acids in any of the strains in carbon limitation.

A previous study reported a change in the fatty acid profile when OKYL029 was cultivated with urea in nitrogen-limiting conditions compared to ammonium sulfate.^25^ Under this condition, we only observed a change in the fatty acid profile for the obese strain OKYL049. However, the observed trends in saturation and fatty acid chain length were inverted compared to the previous study. The previously reported change in fatty acid profile was possibly due to a pH change throughout the shake flask cultivation,^31^ instead of the assimilation of urea per se. Because the ammonium metabolism is acidifying the media, the pH at the end of the cultivation might have been different in cultures with ammonium sulfate compared to urea. Our chemostat cultures were pH-controlled, preventing any influence of different pH. In additon, it is noteworthy that the changes in the fatty acid profile due to the nitrogen source are minor compared to the changes introduced by genetic engineering to increase lipid production (OKYL029 vs OKYL049). Furthermore, these changes (overexpression of DGA1 and Δare1) did not aim to modify the fatty acid profile, while multiple studies have shown that targeted engineering e.g. of the desaturases can massively alter the fatty acid profile.^26^^,^^32^^,^^33^ Our results show that urea can be used as a drop-in nitrogen source instead of ammonium sulfate without massively altering the storage lipid profile.

Urea is converted in a one-enzyme two-step reaction to ammonium and further to glutamate and glutamine. Yeast cells have pathways to regulate nitrogen metabolism, which are often sensed by measuring the cellular glutamine level.^34^ Because we did not observe any significant changes in gene regulation downstream of glutamate, the glutamine level is likely unaffected by ammonium sulfate or urea as nitrogen sources.

Therefore, the minimal overall transcriptomic changes were anticipated and confirmed by only small overlaps of DE genes of different strains and conditions. The number of uncharacterized proteins shows the limitation of omics approaches for unconventional organisms. However, with increasing numbers of studies conducted in *Y. lipolytica* and advancements in protein function prediction, we expect this limitation to reduce gradually.

The near absence of transcriptional induction of any genes directly related to ammonium and urea transport and urea conversion was not anticipated. In *Candida albicans*, the urea transporter DUR3 is highly induced by the presence of urea on a transcriptional level.^35^ In *S. cerevisiae* DUR3 and the urea amidolyase DUR1,2 are induced by the intermediate of the multifunctional enzyme Dur1,2p, allophanate.^36^ We only observed a significant upregulation of one of the four genes homolog to DUR3. This indicates that YALI1_B05609g is the functional homolog of DUR3 with a similar regulation as in related yeast species. However, this upregulation was only observed in nitrogen limitation and not in carbon limitation conditions, in which the initial urea concentration was higher (2.4 g/L versus 0.21 g/L urea in nitrogen limitation). This indicates that the upregulation of DUR3 might be intertwined with the transcriptional response to nitrogen limitation.

Taken together, our physiological data and transcriptomics analysis show that the cell behavior in urea is very comparable to that in ammonium sulfate. Urea is an economically and environmentally attractive nitrogen source that does not acidify the media and requires low base addition during cultivation, further reducing the costs of the process. These findings can open the way for future studies and industrial applications using urea as a sustainable alternative nitrogen source in *Y. lipolytica*.

### Limitation of the study

A limitation of transcriptomic studies in unconventional yeasts is the high amount of uncharacterized genes and proteins. Most of the DE genes identified in this study are uncharacterized. We anticipate that the knowledge gap between conventional and unconventional yeasts will be reduced over the next years, allowing for further insights that are currently out of reach.

In addition, this study is based on transcriptomics analysis and does not take posttranslational modification and regulation into account. Furthermore, we only conducted the study in chemostats with a dilution rate of 0.1. It is possible that the cell behavior could change at different dilution rates.

## STAR★Methods

### Key resources table

**Table:** 

REAGENT or RESOURCE	SOURCE	IDENTIFIER
**Chemicals, peptides, and recombinant proteins**		

Potassium hydroxide	Avantor, VWR	1.05012.5000
Ammonium sulphate	Sigma Aldrich	1012175000
Urea	Sigma Aldrich	U5128-5kg
D(+)-Glucose monohydrate	Avantor, VWR	1.08342.9025
Magnesium sulphate heptahydrate	Merck	10034-99-8
Monopotassium phosphate	Fisher Scientific	7778-77-0
Iron(II) sulfate heptahydrate	Sigma Aldrich	7782-63-0
Zinc sulfate heptahydrate	Sigma Aldrich	7446-20-0
Calcium chloride dihydrate	Sigma Aldrich	10035-04-8
Manganese chloride tetrahydrate	Sigma Aldrich	13446-34-9
Cobalt(II) chloride hexahydrate	Sigma Aldrich	7791-13-1
Copper(II) sulfate pentahydrate	Sigma Aldrich	7758-99-8
Sodium molybdate dihydrate	Sigma Aldrich	10102-40-6
Boric Acid	Sigma Aldrich	10043-35-3
Ethylenediaminetetraacetic acid disodium salt dihydrate	Sigma Aldrich	6381-92-6
d-Biotin	Sigma Aldrich	58-85-5
d-pantothenic acid hemicalcium salt	Sigma Aldrich	137-08-6
Thiamine Hydrochloride Hydrate	Sigma Aldrich	67-03-8
Pyridoxine hydrochloride	Sigma Aldrich	58-56-0
Nicotinic acid	Sigma Aldrich	59-67-6
4-Aminobenzoic acid	Sigma Aldrich	150-13-0
myo-Inositol	Sigma Aldrich	87-89-8
Glyceryl triheptadecanoate	Sigma Aldrich	2438-40-6
Sodium hydroxide	Sigma Aldrich	1310-73-2
Methanol	Sigma Aldrich	67-56-1
Sulphuric acid	Sigma Aldrich	7664-93-9
Hexane	Sigma Aldrich	110-54-3

**Critical commercial assays**		

RNeasy Mini Kit	Qiagen	74104
RNase-Free DNase Set	Qiagen	79254
TruSeq Stranded mRNA	Illumina	20020595

**Deposited data**		

Physiological data	This paper	https://github.com/SysBioChalmers/Yarrowia_Multifactor
RNA-sequencing raw data	This paper	ArrayExpress: E-MTAB-11008
Code and read counts for differential gene expression analysis	This paper	https://github.com/SysBioChalmers/Yarrowia_Multifactor

**Experimental models: Organisms/strains**		

OKYL029	Konzock et al.^25^	N/A
OKYL049	Konzock et al.^24^	N/A
JFYL007	Poorinmohammad et al.^23^	N/A

**Software and algorithms**		

DASware® control 5 version 5.30	Eppendorf	https://www.eppendorf.com/gb-en/eShop-Products/Bioprocess/Bioprocess-Software/DASware-control-p-PF-133797
bcl2fastq_v2.20.0.422	Illumina	https://support.illumina.com/downloads/bcl2fastq-conversion-software-v2-20.html
RNAseq pipeline	Nf-core	https://github.com/nf-core/rnaseq
RStudio version 4.2.1	RStudio	https://www.rstudio.com
OmicsBox version 2.0.24	BioBam	https://www.biobam.com/omicsbox/

**Other**		

DASGIP® Process Computer	Eppendorf	76DGPCR
DASGIP® Bioblock Stirrer Vessels: 1 L Vessel	Eppendorf	76SR0700ODLS
DASGIP® Bioblock	Eppendorf	76DGTBLOCK
DASGIP® MX Modules for TMFC Gas Mixing	Eppendorf	76DGMX44H
DASGIP® MP8 Multi Pump Modules	Eppendorf	76DGMP8X
DASGIP® TC4SC4 for Temperature and Agitation Control	Eppendorf	76DGTC4SC4B
DASGIP® PH4PO4 for Monitoring of pH, DO, Redox and/or Level	Eppendorf	76DGPH4PO4
DASGIP® GA for Exhaust Analysis	Eppendorf	76DGGA1EX
DO sensors	Eppendorf	78108026
pH sensors	Eppendorf	76DGPHMTI220
Thermo Scientific Trace 1310	Thermo Scientific	https://www.thermofisher.com/order/catalog/product/CL2GAS000001
Thermo Scientific ISQ LT	Thermo Scientific	https://www.thermofisher.com/order/catalog/product/ISQEC000IC
ZBFAME column	Phenomenex	7FD-G033-05
UltiMate® 3000	Dionex	N/A
Aminex® HPX-87H ion exclusion column	Bio-Rad	1250140
2100 Bioanalyzer	Agilent Technologies	G2939BA
NovaSeq 6000	Illumina	N/A

### Resource availability

#### Lead contact

Further information and requests for resources and reagents should be directed to and will be fulfilled by the lead contact, Eduard Kerkhoven (eduardk@chalmers.se).

#### Materials availability

Yeast strains used in this study are available from the lead contact with a completed Materials Transfer Agreement.

### Experimental model and subject details

#### Yeast strains

All strains in this study are derived from the *Yarrowia lipolytica* strain ST6512.^37^ ST6512 was derived from the W29 background strain (Y-63746 from the ARS Culture Collection, Peoria, USA; a.k.a. ATCC20460/CBS7504) and has been engineered to harbour a KU70::Cas9-DsdA to allow marker-free genomic engineering using EasyCloneYALI toolbox.^38^ OKYL029 (ST6512 Δmhy1) carries MHY1 deletion to prevent stress-induced hyphae formation.^25^ OKYL049 (ST6512 + E1::pTef1in + DGA1 + tPEX20 Δare1 Δmhy1) is an obese strain carrying DGA1 overexpression and ARE1 deletion to increase TAG accumulation and abolish sterol ester formation^23^; to prevent hyphae formation, MHY1 is deleted. JFYL007 (ST6512 Δmhy1 Δare1 Δlro1 Δdga1 Δdga2) is a low lipid accumulating strain carrying a deletion of ARE1 to abolish sterol ester formation, a deletion of LRO1 (triacylglycerol formation with phospholipids as acyl donors), a deletion of DGA1 (acyl-CoA dependent triacylglycerol synthase), and a deletion of DGA2 (member of the type 1 acyl-CoA:diacylglycerol acyltransferase family) to decrease TAG accumulation; to prevent hyphae formation, MHY1 is also deleted.^24^ Since our strains are derived from the W29/CLIB89 background, our gene annotation follows the YALI1 system, but translation into YALI0/CLIB122) can be done with the S2 table of.^39^

**Table:** 

Strain	Genotype description	Reference
ST6512	W29KU70::Cas9-DsdA	Holkenbrink et al.^38^
OKYL029	ST6512 Δmhy1	Konzock and Norbeck^25^
OKYL049	ST6512 Δmhy1 E1::pTef1in + DGA1 + tPEX20 Δare1	Konzock et al.^23^
JFYL007	ST6512 Δmhy1 Δare1 Δlro1 Δdga1 Δdga2	Poorinmohammad et al.^24^

#### Bioreactor and chemostat cultivation

Chemostat cultivations were performed in DasGip 1-L stirrer-pro vessels (Eppendorf, Jülich, Germany), with a working volume of 500 mL at 28°C. The agitation was set at 600 rpm and aeration with sterile air at 1 vvm (= 30Lh^-1^) to ensure aerobic conditions and monitored with DO probe (Mettler Toledo, Switzerland). pH was monitored with a pH sensor (Mettler Toledo, Switzerland) and maintained at 5.0 ± 0.05 by automatic addition of 2M KOH. Cells were grown in batch using the same media as the chemostat, and their growth was monitored via the CO_2_ exhaust gas. After leaving the exponential growth phase, the constant feed was initiated to obtain steady-state cultivation with a dilution rate of 0.10 h^−1^. The working volume of 500 mL was kept constant using an overflow pump. Samples for transcriptome analysis were taken after at least 4 residence times of steady-state growth. Each condition was cultivated at least in triplicates.

Chemostat cultivations with C/N ratio 3 were performed in delft media containing either 5.28 g/L of ammonium sulphate or 2.4 g/L urea (sterile filtered), 7.92 g/L glucose, 0.5 g/L magnesium sulphate heptahydrate, 3 g/L monopotassium phosphate, 1 mL of trace metals solution, and 1 mL of vitamin solution. pH was set at 5 with KOH.

Chemostat cultivations with C/N ratio 116 were performed in delft media containing either 0.471 g/L of ammonium sulphate or 0.213 g/L urea (sterile filtered), 27.5 g/L glucose, 0.5 g/L magnesium sulphate heptahydrate, 3 g/L monopotassium phosphate, 1 mL of trace metals solution, and 1 mL of vitamin solution. pH was set at 5 with KOH.

Trace metal solution consisted of 3.0 g/L FeSO_4_⋅7H_2_O, 4.5 g/L ZnSO_4_⋅7H_2_O, 4.5 g/L CaCl_2_⋅2H_2_O, 1 g/L MnCl_2_⋅4H_2_O, 300 mg/L CoCl_2_⋅6H_2_O, 300 mg/L CuSO_4_⋅5H_2_O, 400 mg/L Na_2_MoO_4_⋅2H_2_O, 1 g/L H_3_BO_3_, 100 mg/L KI, 19 g/L Na2EDTA⋅2H20.

Vitamin solution consisted of 50 mg/L d-biotin, 1.0 g/L D-pantothenic acid hemicalcium salt, 1.0 g/L thiamin-HCl, 1.0 g/L pyridoxin-HCl, 1.0 g/L nicotinic acid, 0.2 g/L 4-aminobenzoic acid, 25g/L myo-Inositol.

### Method details

#### Lipid extraction and quantification

Samples for lipid extraction and quantification were taken after at least four residence times of steady-state cultivation. The protocol used was previously described.^25^^,^^40^ In short, 1 mL of cell culture was spun down (5 min at 5000 rcf), the supernatant discarded, and the cells washed twice with 1 mL water. The suspension was spun down (5 min at 5000 rcf), the cell pellet resuspended in 100 μL, and dried in a vacuum dry freezer for 1 day. 40 μg of triheptadecanoin (TAG(17:0/17:0/17:0)) was added to the cell pellet as internal standard. 500 μL of 1M NaOH in methanol was added and the samples were vortexed at 1200 rpm at room temperature for 1 h. 80 μL of 49% sulfuric acid was added to neutralize the reaction. FAMEs were extracted by adding 500 μL hexane. Phases were separated by centrifugation (1 min at 10,000 rcf). 200 μL of the upper hexane phase were diluted 1:5 in hexane and 1 μL was analyzed on GC-MS (Thermo Scientific Trace 1310 coupled to a Thermo Scientific ISQ LT with a ZBFAME column (Phenomenex, length: 20 m; Inner Diameter: 0,18 mm; Film Thickness: 0,15 μm)). Lipid content is calculated as lipid content per cell dry weight. Cell dry weight was determined by vacuum filtration of 1 mL of samples on pre-weighed 0.45 μm filter membranes (Sartorius Biolab) followed by microwaving at 125 to 325 W for 15 min and placement in a desiccator for at least 3 days.

#### Extracellular metabolite analysis

Samples for high-performance liquid chromatography (HPLC) were taken after at least four residence times of steady-state cultivation. 1 mL of culture was centrifuged for 5 min at 3000 rcf, and the supernatant was used for high-performance liquid chromatography (HPLC) analysis to quantify extracellular metabolites (acetate, citrate, ethanol, glycerol, glucose, pyruvate, succinate). The HPLC system UltiMate® 3000 (Dionex) was equipped with an Aminex® HPX-87H ion exclusion column (Bio-Rad). 5 mM H_2_SO_4_ was used as eluent at a flow rate of 0.6 mL/min. Glucose was quantified using a refractive index detector (Shodex ri-101).

#### RNA extraction and sequencing

Samples for RNA extraction were taken after at least four residence times of steady-state cultivation by rapidly withdrawing 10 mL of culture and injecting it into a 50 mL falcon tube with ca. 35 mL of crushed ice. The samples were immediately centrifuged at 4000 rcf for 5 min at 4°C. The supernatant was discarded, the pellet was resuspended in ice-cold water, transferred into 2 mL reaction tubes, and centrifuged at 4000 rcf for 5 min at 4°C. The supernatant was removed, and the open tubes were tapped on paper towels to remove the remaining residue. Dry samples were immediately frozen in liquid nitrogen and stored at −80°C until further analysis.

RNA was extracted using the RNeasy Mini Kit (QIAGEN). The DNA was removed from the sample using RNase-Free DNase Set (QIAGEN). The quality of purified RNA samples was analyzed with a 2100 Bioanalyzer (Agilent Technologies, Inc., Santa Clara, CA) and the purified RNA was stored at −80°C until further analysis.

The RNA library was constructed using Illumina TruSeq Stranded mRNA (poly-A selection) and samples were sequenced on NovaSeq6000 (NovaSeq Control Software 1.7.5/RTA v3.4.4) with a 151 nt(Read1)-10nt(Index1)-10nt(Index2)-151nt(Read2) setup using 'NovaSeqXp' workflow in 'S4′ mode flowcell. The Bcl to FastQ conversion was performed using bcl2fastq_v2.20.0.422 from the CASAVA software suite. The quality scale used was Sanger/phred33/Illumina 1.8+..

### Quantification and statistical analysis

#### Differential gene expression analysis

The raw reads were processed with the NGI RNAseq Pipeline (https://github.com/nf-core/rnaseq.git), version 3.5. The *Y. lipolytica* strain CLIB89(W29) reference genome was used to map the reads (assembly GCA_001761485.1). Differential gene expression analysis was performed with limma,^41^ and adjusted p-values were calculated according to the method of Benjamini–Hochberg. OmicsBox (https://www.biobam.com/omicsbox) was used to generate gene sets by blasting *Y. lipolytica* exons against the RefSeq non-redundant proteins database using BlastX algorithm. A total of 31.421 GO terms were annotated to 5629 genes. Gene set analysis was performed using the R package PIANO (Platform for Integrative Analysis of Omics),^42^ using gene levels statistics, ignoring gene-sets containing less than five genes or more than 500 genes. The code used for the analysis is available on GitHub (https://github.com/SysBioChalmers/Yarrowia_Multifactor).

### Data and code availability

The raw RNA-seq data are deposited in ArrayExpress (ArrayExpress: E-MTAB-11008).

The code and data used for the analysis were deposited to the Github repository and are available at https://github.com/SysBioChalmers/Yarrowia_Multifactor.

## Data Availability

• RNA-seq data have been deposited on ArrayExpress (ArrayExpress: E-MTAB-11008) and are publicly available as of the date of publication. Accession numbers are listed in the key resources table. • All original code has been deposited on GitHub (https://github.com/SysBioChalmers/Yarrowia_Multifactor) and is publicly available as of the date of publication. DOIs are listed in the key resources table. • Any additional information required to reanalyze the data reported in this paper is available from the lead contact upon request.
