# Supplementary material for: Urea is a drop-in nitrogen source alternative to ammonium sulphate in Yarrowia lipolytica
Source: iScience. 2022 Dec 1;25(12):105703. doi: 10.1016/j.isci.2022.105703 (PMC9772842; doi:10.1016/j.isci.2022.105703)

iScience, Volume *25*

Supplemental information

Urea is a drop-in nitrogen source alternative to ammonium sulphate in *Yarrowia lipolytica*

Oliver Konzock, Simone Zaghen, Jing Fu, and Eduard J. Kerkhoven

Supplementary

Table S1: Genes related to urea metabolism and their annotations from different sources. Related to **Figure 4**.

Gene	Yali1	Yali0	Yali Model *	Annotation **	Uniprot Protein Name ***
DUR3	YALI1_B05609g	YALI0B04202g	Yes	similar to uniprot|P33413 Saccharomyces cerevisiae YHL016c DUR3 urea transport protein	Uncharacterized protein
DUR3	YALI1_C22751g	YALI0C15807g	Yes	similar to uniprot|P33413 Saccharomyces cerevisiae YHL016c DUR3 urea transport protein	Uncharacterized protein
DUR3	YALI1_E33888g	YALI0E28622g	No	similar to uniprot|P33413 Saccharomyces cerevisiae YHL016c DUR3 urea transport protein	Urea active transporter 1
DUR3	YALI1_E39848g	YALI0E33583g	No	similar to uniprot|P33413 Saccharomyces cerevisiae YHL016c DUR3 urea transport protein singleton	Sodium:solute symporter family-domain-containing protein
DUR1_2	YALI1_B19217g	YALI0B14619g	Yes	weakly similar to uniprot|P32528 Saccharomyces cerevisiae YBR208c DUR1_2 urea amidolyase	Carbamoyl-phosphate synthase L chain, ATP binding domain-domain-containing protein
DUR1_2	YALI1_E08620g	YALI0E07271g	Yes	similar to uniprot|P32528 Saccharomyces cerevisiae YBR208c DUR1_2 urea amidolyase	Urea amidolyase
DUR1_2	YALI1_E41754g	YALI0E35156g	Yes	similar to uniprot|P32528 Saccharomyces cerevisiae YBR208c DUR1_2 urea amidolyase P6.9.f5.1	Uncharacterized protein
MEP1,2,3	YALI1_A02653g	YALI0A02244g	No	weakly similar to uniprot|P41948 Saccharomyces cerevisiae YNL142w MEP2 high affinity low capacity ammonia permease	Ammonium_transp domain-containing protein
MEP1,2,3	YALI1_A20201g	YALI0A19228g	No	similar to uniprot|Q9P7F3 Schizosaccharomyces pombe Ammonium transporter	Ammonium transporter
MEP1,2,3	YALI1_E32180g	YALI0E27203g	Yes	similar to uniprot|P41948 Saccharomyces cerevisiae YNL142w MEP2	Ammonium transporter
MEP1,2,3	YALI1_F17337g	YALI0F12925g	Yes	similar to uniprot|P41948 Saccharomyces cerevisiae YNL142w MEP2 high affinity low capacity ammonia permease	Ammonium transporter
MEP1,2,3	YALI1_F22568g	YALI0F16896g	Yes	similar to uniprot|P40260 Saccharomyces cerevisiae YGR121c MEP1 ammonia permease of high capacity and moderate affinity or uniprot|P53390 Saccharomyces cerevisiae YPR138c MEP3 low affinity high capacity ammonium permease	Ammonium transporter

MEP1,2,3	YALI1_B18292g	YALI0B13794g	No	similar to uniprot|Q9P7F3 Schizosaccharomyces pombe Ammonium transporter	Ammonium transporter
PMA1	YALI1_B28659g	YALI0B22066g	Yes	highly similar to uniprot|P05030 Saccharomyces cerevisiae YGL008c PMA1 H+-transporting P-type ATPase major isoform plasma membrane	Plasma membrane ATPase
GDH1	YALI1_F23664g	YALI0F17820g	Yes	similar to Saccharomyces cerevisiae GDH3 (YAL062W) and GDH1 (YOR375C)%3B ancestral locus Anc_7.1, highly similar to uniprot|P39708 Saccharomyces cerevisiae YAL062w GDH3 NADP-glutamate dehydrogenase or uniprot|P07262 Saccharomyces cerevisiae YOR375c GDH1 glutamate dehydrogenase (NADP+)	Glutamate dehydrogenase
GDH2	YALI1_E11943g	YALI0E09603g	Yes	similar to Saccharomyces cerevisiae GDH2 (YDL215C)%3B ancestral locus Anc_2.68, no similarity, similar to uniprot|P33327 Saccharomyces cerevisiae YDL215c GDH2 NAD-specific glutamate dehydrogenase (NAD)	NAD-specific glutamate dehydrogenase
GLN1	YALI1_F00821g	YALI0F00506g	Yes	similar to Saccharomyces cerevisiae GLN1 (YPR035W)%3B ancestral locus Anc_7.449, highly similar to uniprot|P32288 Saccharomyces cerevisiae YPR035w GLN1 glutamate--ammonia ligase and DEHA0G20317g Debaryomyces hansenii IPF 5106.1	Glutamine synthetase
GLT1	YALI1_B26112g	YALI0B19998g	Yes	similar to uniprot|Q12680 Saccharomyces cerevisiae YDL171c GLT1 glutamate synthase (NAPDPH) (GOGAT,similar to Saccharomyces cerevisiae GLT1 (YDL171C)%3B ancestral locus Anc_7.361	Glutamate synthase (NADH)

* Yes/No if the reaction is included in the Yali model, https://github.com/SysBioChalmers/Yarrowia_lipolytica_W29-GEM (V4.1.2)

** Annotation according to https://doi.org/10.1371/journal.pone.0162363 S2 Table

*** Protein names associated to Yali1 gene name by Uniprot 28.02.2022

Table S2: Genes and their protein functions found in most relevant clusters of the network plot (related to **Figure 3**). Circle and square indicate genes that are down or up-regulated in urea compared to ammonium sulphate. Protein functions were retrieved from UniProt (05.05.2022).

		Gene	Protein function
**A**	■	YALI1_A01298g	Endoplasmic reticulum-based factor for assembly of V-ATPase-domain-containing protein
	■	YALI1_C01505g	Uncharacterized protein
**B**	■	YALI1_B05639g	Uncharacterized protein
	■	YALI1_D07252g	Uncharacterized protein
	■	YALI1_D07257g	Uncharacterized protein
**C**	●	YALI1_E31154g	Uncharacterized protein
	●	YALI1_F13170g	S-(hydroxymethyl)glutathione dehydrogenase (EC 1.1.1.284)
	■	YALI1_C01520g	Uncharacterized protein
**D**	●	YALI1_A01394g	Cation_ATPase_N domain-containing protein
	●	YALI1_A07161g	Uncharacterized protein
	●	YALI1_E12893g	Amino acid permease-domain-containing protein
	●	YALI1_E12919g	Uncharacterized protein
	●	YALI1_F21989g	M20_dimer domain-containing protein
	■	YALI1_B05609g	Uncharacterized protein
**E**	●	YALI1_A16918g	Uncharacterized protein
	●	YALI1_A16944g	Uncharacterized protein
	●	YALI1_A16952g	Uncharacterized protein
	●	YALI1_B30303g	Uncharacterized protein
	●	YALI1_D08621g	Uncharacterized protein
	●	YALI1_E21535g	Uncharacterized protein
	●	YALI1_E21552g	Uncharacterized protein
	●	YALI1_E21555g	Uncharacterized protein
	●	YALI1_E22503g	Uncharacterized protein
	●	YALI1_E24287g	Uncharacterized protein
	●	YALI1_E27406g	WD40-repeat-containing domain protein
	●	YALI1_E27625g	Uncharacterized protein
	●	YALI1_F11635g	NUDIX hydrolase domain-like protein
	●	YALI1_F21676g	Uncharacterized protein
	■	YALI1_C29576g	Uncharacterized protein

Figure S1: Addition of base to the steady-state cultivation in C/N ratio 3 and 116. Volume (mL) of base (2 M KOH) added per residence time (dilution rate 0.1, 10 hours residence time) during steady-state cultivation, normalized to biomass (g). Dots and error bars represent the average and standard deviation of the replicates, respectively. AS in the legend refers to ammonium sulphate. Related to section "Cell physiology does not change with urea compared to ammonium sulphate as a nitrogen source" and **Figure 4**.

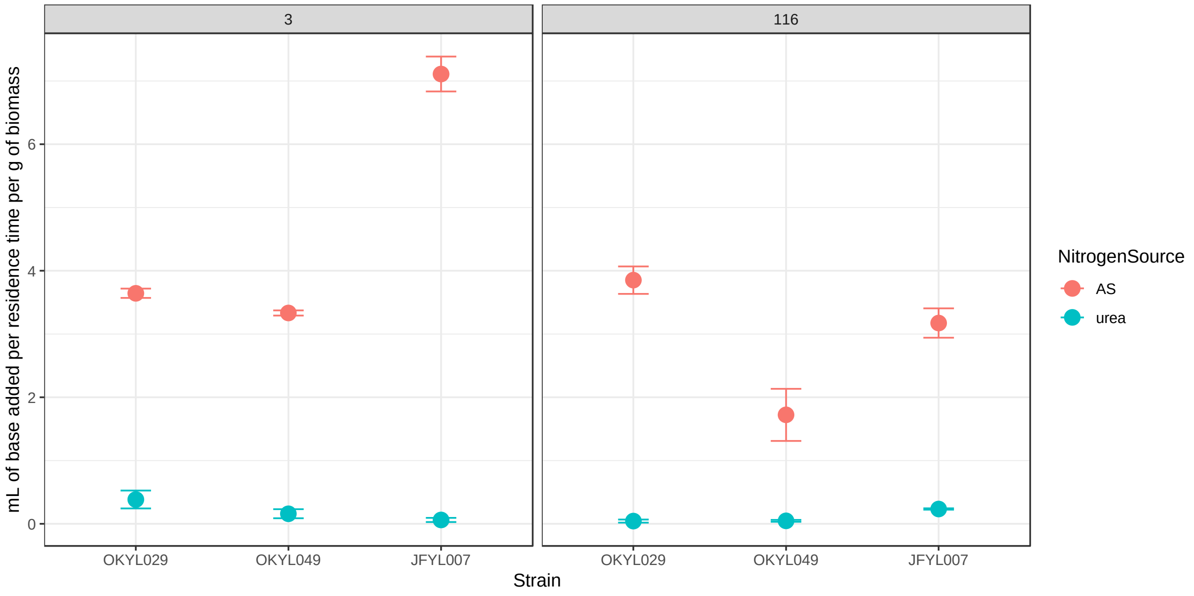

Figure S2: Heat map of GO terms from consensus gene set analysis (GSA). Reported are the GO terms related to biological process. Cells' colours indicate the rank score of the consensus GSA: red indicates a rank score close to 1 (important GO term), white a rank score of 25 (less important), and grey indicates that the comparison did not identify the GO term. U vs AS in the column labels refers to the urea versus ammonium sulphate comparison. Related to STAR methods section "Code and read counts for differential gene expression analysis" and discussed in section "Gene Ontology Analysis revealed no coherent systemic response to the nitrogen source"

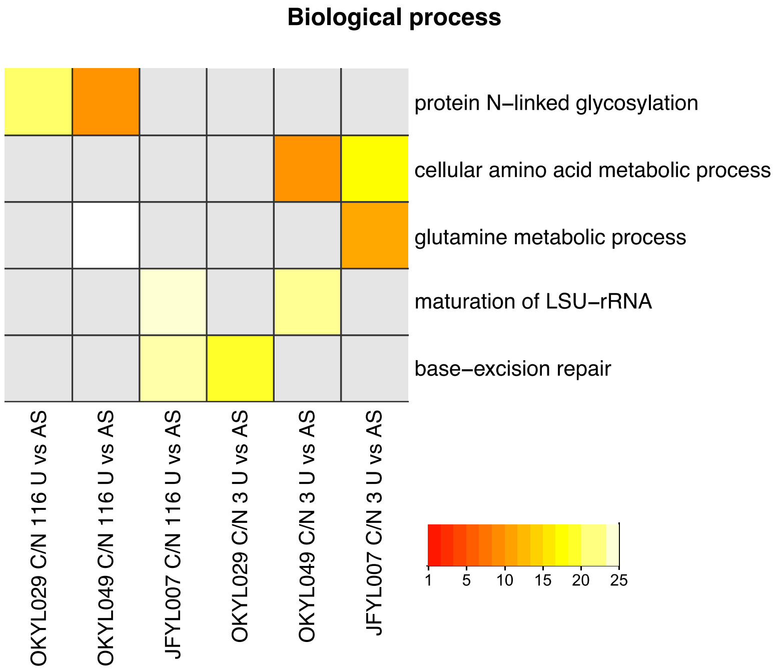

Figure S3: Heat map of GO terms from consensus gene set analysis (GSA). Reported are the GO terms related to molecular function. Cells' colours indicate the rank score of the consensus GSA: red indicates a rank score close to 1 (important GO term), white a rank score of 25 (less important), and grey indicates that the comparison did not identify the GO term. U vs AS in the column labels refers to the urea versus ammonium sulphate comparison. Related to STAR methods section "Code and read counts for differential gene expression analysis" and discussed in section "Gene Ontology Analysis revealed no coherent systemic response to the nitrogen source".

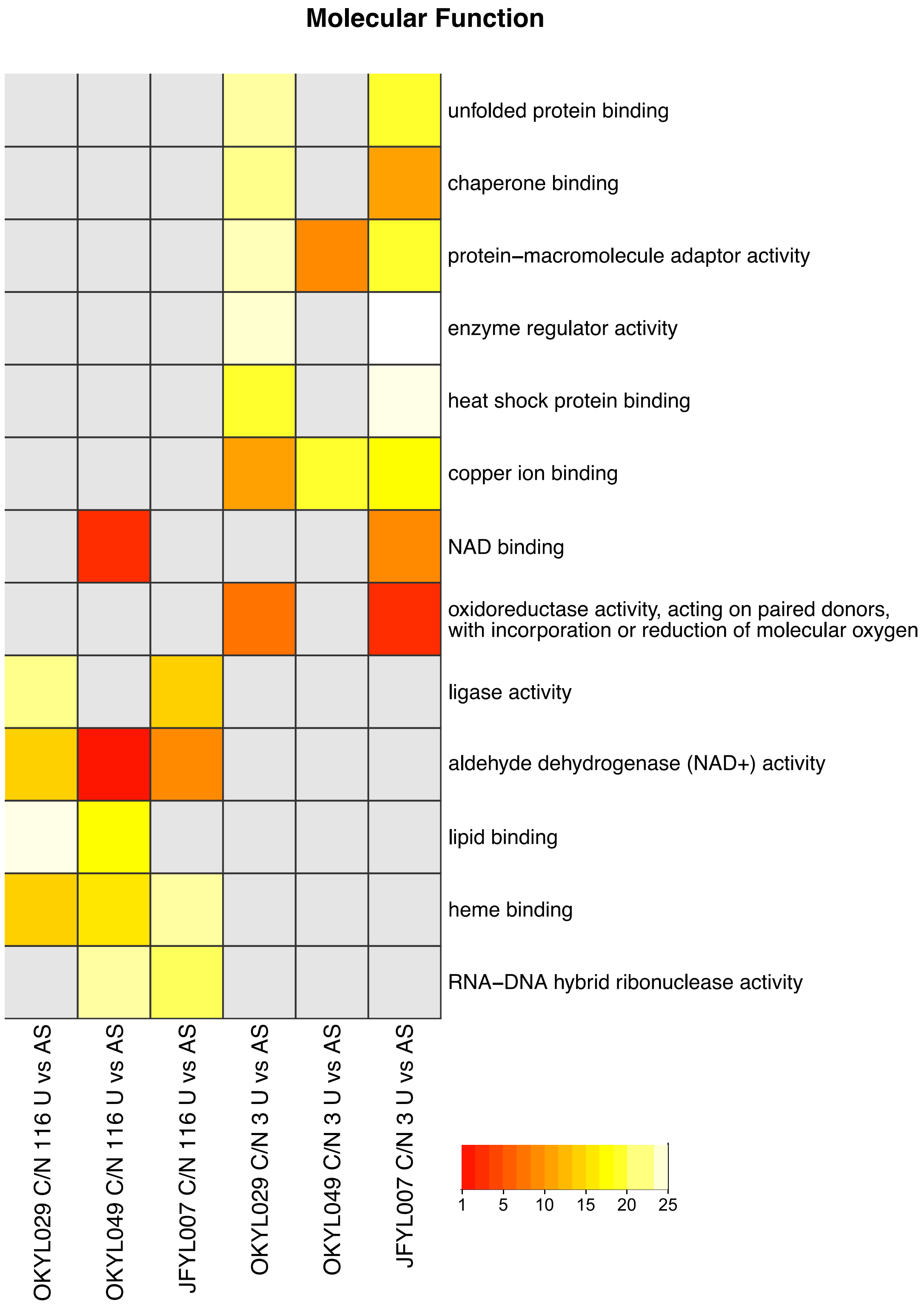

Figure S4: Heat map of GO terms from consensus gene set analysis (GSA). Reported are the GO terms related to cellular component. Cells' colours indicate the rank score of the consensus GSA: red indicates a rank score close to 1 (important GO term), white a rank score of 25 (less important), and grey indicates that the comparison did not identify the GO term. U vs AS in the column labels refers to the urea versus ammonium sulphate comparison. Related to STAR methods section "Code and read counts for differential gene expression analysis" and discussed in section "Gene Ontology Analysis revealed no coherent systemic response to the nitrogen source".

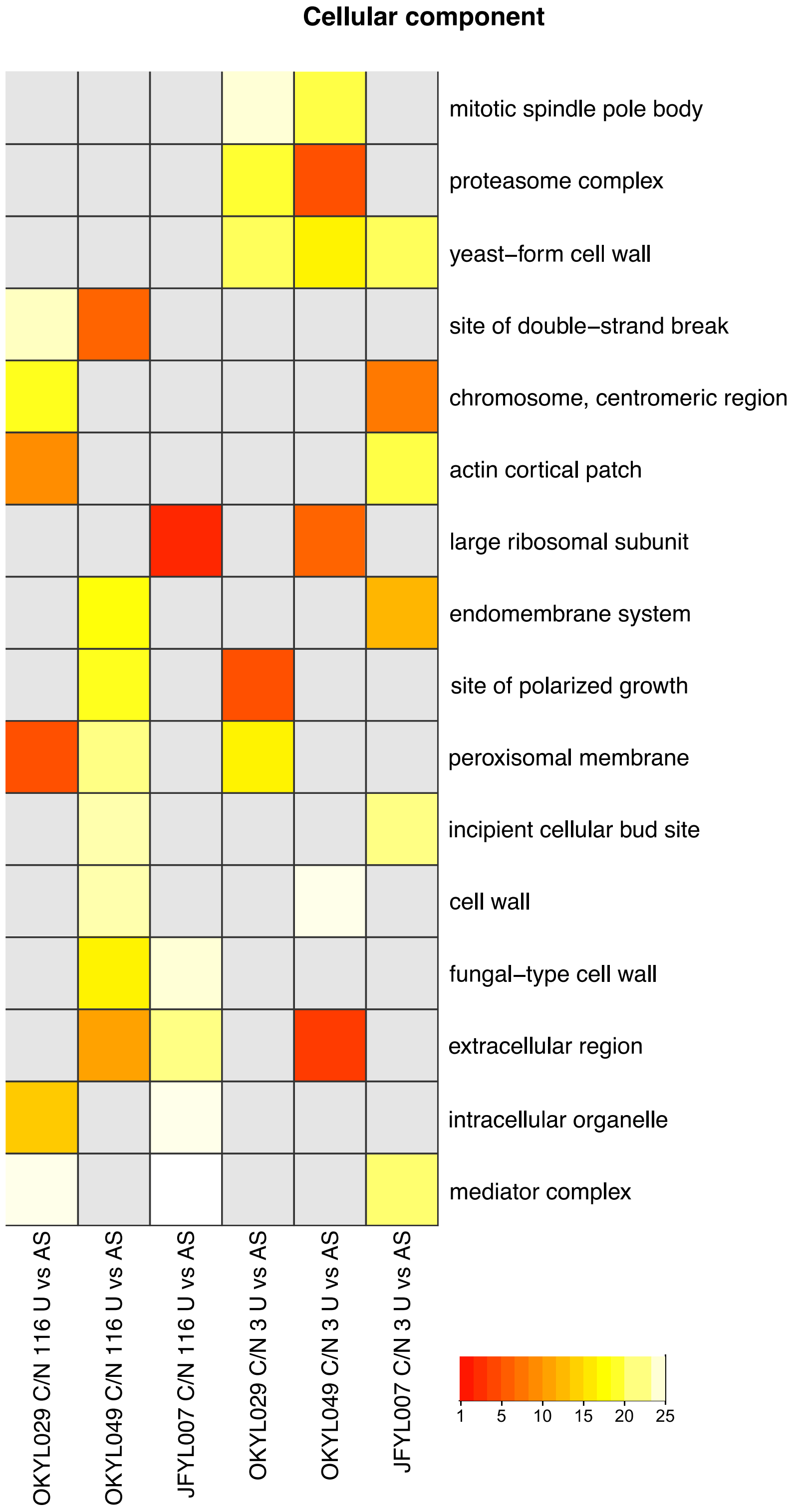

Supplement: Document S1. Figures S1–S4 and Tables S1 and S2 [file mmc1.pdf]
